# P-2201. A Comprehensive Kidney Biomarker Panel to Assess Kidney Health after Hepatitis C Direct Acting Antiviral Therapy in Persons with HCV monoinfection and HCV/HIV coinfection

**DOI:** 10.1093/ofid/ofae631.2355

**Published:** 2025-01-29

**Authors:** Lila A Perrone, Yifei Ma, Kasey Campos, Warren Tse, Alexandra Tien-Smith, Heather Freasier, Rebecca Scherzer, Michelle Estrella, Michael Shlipak, Phyllis C Tien

**Affiliations:** Stanford University, San Francisco, California; University of California, San Francisco, San Francisco, California; UCSF/San Francisco VA Healthcare System, San Francisco, California; UCSF/San Francisco VA Healthcare System, San Francisco, California; UCSF/San Francisco VA Healthcare System, San Francisco, California; UCSF/SF VA Healthcare System, San Francisco, California; UCSF/San Francisco VA Healthcare System, San Francisco, California; UCSF/San Francisco VA Healthcare System, San Francisco, California; UCSF/San Francisco VA Healthcare System, San Francisco, California; University of California, San Francisco, San Francisco, California

## Abstract

**Background:**

Chronic Hepatitis C virus (HCV) infection is associated with greater risk of chronic kidney disease and proteinuria. We examined effects of 12 weeks of elbasvir/grazoprevir, an HCV NS5A inhibitor/NS3/4A Protease Inhibitor on glomerular filtration (creatinine, cystatin C), injury (albuminuria), and 9 novel kidney tubule biomarkers in adults with HCV infection over a 24-week period.

**Figure.**
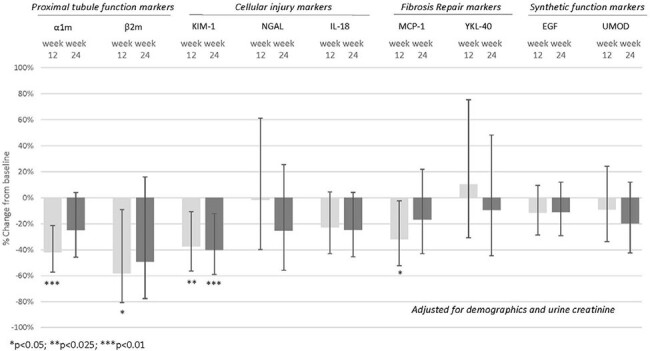
Percent Change (95% Confidence Interval) from Baseline of Urine Kidney Tubule Markers 24 weeks after initiating Elbasvir/Grazoprevir in Patients with HCV

**Methods:**

We included 36 participants with HCV (7 with HIV coinfection) from the San Francisco Bay Area. Urine biomarkers included kidney tubule function (α-1-microglobulin [α1m], β-2-microglobulin [β2m]) and injury (kidney injury marker-1 [KIM-1], neutrophil gelatinase-associated lipocalcin [NGAL], (interleukin-18 [IL-18]), fibrosis/repair (monocyte chemoattractant protein 1 [MCP-1], chitinase-3-like protein-1 [YKL-40]) and synthetic function (epidermal growth factor [EGF], uromodulin [UMOD]). Biomarkers were measured at baseline, wk 12 (end of treatment), and week 24 (time of assessment for sustained virologic response [SVR]). We used linear mixed regression models with time as the main predictor to assess changes in kidney health, adjusting for demographics and urine creatinine.

**Results:**

Over two thirds identified as Black/African American;89% were men and median age was 62 years. All but 2 achieved an SVR and 17% had advanced liver fibrosis using the FIB-4 serum marker. Median (interquartile range) for eGFR_cr,_ eGFR_cys,_ and urine albumin to creatinine ratio (ACR) were 82(63-100 ml/min),74(58-87 ml/min), and 10.4(4.9-24 mg/g), respectively at baseline. The percent (%) change from baseline for eGFR_cr_ and ACR at weeks 12 and 24 was not statistically significant; eGFR_cys_ was -11.4% lower (95%CI:-18%,-4.6%) at week 24. After 12 weeks of treatment, there were significant reductions in α1m, β2m, KIM-1, and MCP-1, with smaller reductions seen in most other markers (Figure). At week 24, these reductions were sustained for KIM-1.

**Conclusion:**

Our findings suggest that HCV treatment with elbasvir/grazoprevir improves proximal tubule function and reduces cellular injury and fibrosis. Further research is needed to understand longer-term trends in kidney health following HCV cure.

**Disclosures:**

Michelle Estrella, MD, Astra Zeneca: Board Member|Bayer: Grant/Research Support|Boehringer Ingelheim: Board Member Michael Shlipak, MD, Astra-Zeneca: Honoraria|Bayer: Grant/Research Support|Bayer: Honoraria|Boehringer Ingelheim: Honoraria Phyllis C. Tien, MD, MSc, Merck: Grant/Research Support

